# Randomization in substance abuse clinical trials

**DOI:** 10.1186/1747-597X-1-6

**Published:** 2006-02-06

**Authors:** Sarra L Hedden, Robert F Woolson, Robert J Malcolm

**Affiliations:** 1 Department of Biostatistics, Bioinformatics and Epidemiology (DB^2^E), Medical University of South Carolina, Cannon Place, Cannon Street, Charleston, SC. 29425, USA; 2 Center for Drug and Alcohol Programs (CDAP), Medical University of South Carolina, Institute of Psychiatry, President Street, Charleston, SC, 29425, USA

## Abstract

**Background:**

A well designed *randomized *clinical trial rates as the highest level of evidence for a particular intervention's efficacy. Randomization, a fundamental feature of clinical trials design, is a process invoking the use of probability to assign treatment interventions to patients. In general, randomization techniques pursue the goal of providing objectivity to the assignment of treatments, while at the same time balancing for treatment assignment totals and covariate distributions. Numerous randomization techniques, each with varying properties of randomness and balance, are suggested in the statistical literature. This paper reviews common randomization techniques often used in substance abuse research and an application from a National Institute on Drug Abuse (NIDA)-funded clinical trial in substance abuse is used to illustrate several choices an investigator faces when designing a clinical trial.

**Results:**

Comparisons and contrasts of randomization schemes are provided with respect to deterministic and balancing properties. Specifically, Monte Carlo simulation is used to explore the balancing nature of randomization techniques for moderately sized clinical trials. Results demonstrate large treatment imbalance for complete randomization with less imbalance for the urn or adaptive scheme. The urn and adaptive randomization methods display smaller treatment imbalance as demonstrated by the low variability of treatment allocation imbalance. For all randomization schemes, covariate imbalance between treatment arms was small with little variation between adaptive schemes, stratified schemes and unstratified schemes given that sample sizes were moderate to large.

**Conclusion:**

We develop this paper with the goal of reminding substance abuse researchers of the broad array of randomization options available for clinical trial designs. There may be too quick a tendency for substance abuse researchers to implement the fashionable urn randomization schemes and other highly adaptive designs. In many instances, simple or blocked randomization with stratification on a major covariate or two will accomplish the same objectives as an urn or adaptive design, and it can do so with more simply implemented schedules and without the dangers of overmatching. Furthermore, the proper analysis, fully accounting for the stratified design, can be conducted.

## Background

### Introduction

Emerging in the 1980's, evidence-based medicine is quickly becoming the standard for classification of health care outcome research [1]. The classification relies on a hierarchy of evidence for efficacy where *randomized *clinical trials rate as the highest level of evidence for a particular intervention's efficacy [2]. Specifically, randomization provides the basis for the unbiased comparison of treatment effect and provides validity for tests of statistical significance [3]. Therefore, the selection of appropriate randomization techniques for a clinical trial is a fundamental feature of the hierarchy by which a clinical trial is judged for evidence of efficacy.

Multiple randomizations schemes have been suggested for use in the statistical literature. Given the many schemes provided in the literature, a daunting task for the substance abuse researcher is to differentiate between schemes in order to choose the most optimal randomization scheme for a particular clinical trial. A goal of this paper is to review and elaborate on the properties associated with several randomization schemes commonly considered in designing substance abuse clinical trials. We also wish to present distinguishing features of these randomization schemes in order to aid the substance abuse researcher in the selection of an appropriate randomization scheme.

Simplistically, randomization schemes can be differentiated as either restricted or unrestricted. Whereas unrestricted schemes have no constraints imposed on the random allocation of treatments, restricted schemes impose "balancing" restrictions on the probability of treatment allocation, e.g. equal numbers of patients per treatment group. Balance restrictions generally consist of two types of restrictions, those that impose balance on treatment assignment throughout the length of the trial in order to achieve equal numbers of subjects within each treatment assignation, and those that impose covariate balance between treatment groups, e.g. an equal distribution of males and females across treatment groups.

A variety of randomization schemes have been described in the statistical literature; however, common schemes utilized in the substance abuse literature are: *complete, simple, permuted block*, *urn *and *covariate adaptive *randomization. *Complete *randomization, unlike restricted forms of randomization, does not impose balance restrictions of the total number of participants assigned to each treatment. In contrast, the *simple*, *permuted block*, the *urn *and the *covariate adaptive *randomization are examples of restricted designs, which impose balance restriction on the probability of treatment allocation throughout the length of a trial. *Urn *randomization in contrast to *simple *or *permuted block *randomization is dynamic; the probability of treatment assignment changes dependent on the degree of treatment imbalance throughout the course of the trial. The *simple*, *permuted block *and *urn *randomization schemes may be implemented with a further restriction, namely, *stratification*, which imposes covariate balance restriction between treatment groups. *Covariate adaptive *randomization seeks to improve balance in both the final number of subjects assigned to treatment and also the covariate distribution between treatment arms. While *stratification *requires parsimony in the selection of stratification factors, *covariate adaptive *randomization utilizes all covariates known a priori to affect treatment to achieve distributional balance.

Recently, complex schemes such as urn or adaptive forms of randomization have been identified as more modern methods of randomization [4,5]. Whereas these schemes do have the advantage of ensuring balance, it should be reiterated that these schemes might be best used in clinical trials of small sample size (n < 200) where major imbalance could occur with higher probability [3]. However, with moderate to large trials where imbalances have less probability of occurrence, a simple and easily implemented scheme may be more suitable. Furthermore, selection of a randomization scheme in favor of simplicity (e.g. fewer restrictions) is warranted due to restricted methods of randomization requiring more complex methods of analysis.

Although restricted randomization schemes are used in a variety of clinical settings at the design stage of a study, the restrictions are often ignored at the analysis stage of the study. Given that assumptions of parametric models are met, randomization restrictions may be ignored in the analysis and the population model may be used as a method of inference. However, assumptions of population models are not always met and the source of heterogeneity in the data may be hard to determine. Often randomization restrictions are not considered or incorporated into the statistical analysis plan, especially for the most complex schemes such as urn or covariate adaptive randomization where the effects of ignoring the randomization in the analysis are difficult to assess [3]. The statistical literature would argue that the analysis of randomized clinical trials should consider the restrictions imposed by the type of randomization scheme designated at the design stage of the study [3,6-11]. The design and analysis of a clinical trial are not separate entities; rather the analysis is an extension of the design of a clinical trial.

In the sequel, several randomization schemes are both computationally and methodologically detailed. Computations for the randomization techniques are denoted in the Appendices. Comparisons and contrasts of randomization schemes are provided with respect to deterministic and balancing properties. Also, a particular application in substance abuse research is used to illustrate the balancing properties of several randomization schemes. Finally, we will discuss the impact on analysis that balance restrictions entail in order to aid the substance abuse researcher in developing a statistically sound design and analysis for a randomized clinical trial.

We develop this paper with the goal of reminding substance abuse researchers of the broad array of randomization options available for clinical trial designs. There may be too quick a tendency for substance abuse researchers to implement the fashionable urn randomization schemes and other highly adaptive designs. In many instances, simple randomization with stratification on a major covariate or two will accomplish the same objectives as an urn design, and it can do so with more simply implemented schedules and without the dangers of overmatching. Furthermore, the proper analysis, fully accounting for the stratified design, can be conducted. A fundamental message is that simplistic, stratified randomization designs are an efficient class of designs for modest size trials.

### Randomization techniques

In this section we outline several key factors in choosing from a variety of randomization techniques for a substance abuse trial. These include techniques used for both treatment balance (simple, blocked, and urn) and covariate balance (stratification and minimization) within treatments. It should be noted that several of the techniques might be used in combination to obtain both total treatment balance and covariate balance across treatments. For example, stratification may be used with simple, blocked or urn randomization. Covariate adaptive randomisation; by itself, is a randomization technique that achieves both total treatment balance and covariate balance.

### Stratification

In order to achieve balanced treatment distributions within a prognostic factor, subjects may be stratified into groups, and then randomized to treatment within these groups. For example, in clinical trials of cocaine dependence gender is thought to affect outcome independently of treatment; therefore, gender may be used as a stratifying factor. Specifically, a randomization schedule for males, and a separate one for females, are prepared and used to ensure treatment balance exists for each gender stratum. Hence, within each gender, the desired treatment balance is maintained.

More generally, stratification is a technique which partitions patients into mutually exclusive subsets defined by initial covariates thought to influence response and this stratification is utilized to reduce accidental bias [10]. Accidental bias is defined as bias that occurs when nuisance factors that may be known or unknown to the experimenter systematically affect the experimental units [12]. Therefore, stratification is a method used to achieve distributional balance of covariates between treatment groups (or balanced treatment assignments within each level of a stratum) that are expected a priori to influence outcome.

The purpose of stratified randomization is to provide increased confidence that compared groups are similar with respect to known prognostic factors; therefore, differences in endpoints may be attributed to treatment [13] or, of course, chance. Potential advantages of stratification include reduction of Type I error, reduction of Type II error, and increased estimation efficiency [13-15].

Type I error, we know, is defined as declaring a difference in outcomes between treatment groups when in fact no difference exists. In trials with up to 400 patients, statistical studies demonstrate that stratification helped in reducing the probability of type I error [13,14]

Another advantage of stratification includes increases in power of statistical tests. Power, we know, is defined as the ability to detect a difference in treatment groups when a difference exists. In statistical studies of 100 subjects, power increases of up to 12% have been demonstrated when both stratified randomization with adjusted analysis were utilized [13,15].

Although stratification has many advantageous properties, stratification variables and levels within each stratum must be limited to represent only the most important variables and levels. Use of a minimal number of factors in creating the stratification simplifies the randomization and trial administration [13]. Over-stratification can lead to imbalances in overall treatment allocations because large numbers of strata can produce small patient numbers within strata. For example, imbalances in prognostic variables may occur between treatment groups in permuted block designs (defined in the next section) as a result of incomplete block filling. As long as the number of strata for a particular trial is small, there is no disadvantage for stratification; however, over-stratification should be avoided.

In summary we would like the distribution of prognostic factors and confounders to be equalized between treatment arms in order to minimize treatment estimation bias caused by any imbalance. Stratification is a common way of neutralizing such potential covariate imbalance. It attempts to balance treatment groups within the variable levels or strata of each prognostic factor. In order to reduce problems associated with a large selection of strata, only those prognostic factors assumed to have the most powerful effect on outcome are utilized as stratification factors

### Complete & simple randomization

Randomization with no restrictions imposed on the nature of the allocation sequence with the exception of pre-specification of the total sample size is referred to as simple randomization [3]. As an example, simple randomization occurs when the total sample size is exactly pre-specified whereby a randomly chosen subset of n/2 out of n subjects is allocated to treatment 1 and the remaining n/2 subjects are allocated to treatment 2.

Complete randomization is the only scheme without restrictions; that is, treatment assignments are unbiased or random. Essentially with two treatments, each successive patient has a 1/2 probability of receiving treatment 1, and a 1/2 probability of being assigned to treatment 2. This is so irrespective any treatment balances or imbalances up to that point in the trial. Therefore, an important property of complete randomization is the minimization of the determinism of the treatment selection process.

However, given there are no restrictions on the number of patients assigned to each treatment, large imbalances may occur. For example, if treatment 1 or treatment 2 is to be assigned to each of 10 patients, with complete randomization it is possible that any from 0 to 10 of the patients will be assigned treatment 1. For this example with a total of 10 subjects, the probability of perfect balance (5 subjects assigned to treatment one and 5 subjects assigned to treatment 2) is only .2461. That is, imbalance will occur 75% of the time by chance alone. Therefore, a major problem of complete randomization is the non-zero probability of imbalance in treatment assignation, in addition to the smaller, but still non-zero, probability of major imbalance. If we define major imbalance as greater than a 20% difference in treatment assignment, then the probability of major imbalance given 10 subjects is .0216.

As noted, a property of complete randomization is the minimization of selection bias due to the equal probability of correct guess for each sequential treatment assignment. Since complete randomization assigns treatment with equal probability, investigators, staff and subjects are unable to guess the treatment group to which they will be assigned; thus, minimizing the determinism of the assignment. As observed earlier, a major problem of complete randomization is the non-zero probability of imbalance in treatment assignation, in addition to the smaller, but still non-zero, probability of major imbalance. Imbalances may affect the statistical properties of the study including a decrease in the precision of the estimators for treatment group differences and a decrease in the power of a statistical test. Moderate to large imbalances in the number of subjects assigned to treatment may affect the power of a statistical test. Generally, in clinical trials with sample sizes greater than 200 it is highly unlikely that the power of a test will be largely affected by treatment imbalances [3].

Imbalance in the number of subjects assigned to treatment may affect the power of a statistical test, but will not bias the estimate of the treatment effect [16]. Biased treatment effect estimates can occur in the presence of covariate imbalance. The goal of many randomization schemes is, therefore, not only to minimize the imbalance, which occurs when the numbers of subjects assigned to treatment are not equal, but also to minimize the imbalance that occurs when the subjects within treatment groups differ with respect to covariates. Unfortunately, complete randomization does not control for covariate imbalance. Also, stratification cannot be used with complete randomization to improve covariate imbalance because the probability of imbalance for the complete randomization scheme is equal to the probability of imbalance for the stratified complete randomization scheme [3]. However, perfect covariate balance can be attained using both stratification and simple randomization. That is, participants may be grouped by covariates and randomized within strata given the restriction that the total number of people assigned to each treatment within a particular stratum is equal.

### Restricted randomization: permuted blocks as an example

Certain methods of restricted randomization attempt to correct for the probability of treatment imbalance by imposing the restriction that the final allocation is exactly equal between treatment groups. Simple randomization, mentioned previously, has the restriction that the total number of people assigned to each treatment within a particular stratum is equal. However, the balance in treatment numbers is not obtained until the total sample size is reached. Randomizing participants within sequential blocks is an example of a design, which improves balance in the number of treatment assignments throughout the length of the study [3,16].

By imposing balance restriction at interval periods, this block design ensures that the number of subjects assigned to treatment is balanced throughout the course of the trial. However, block designs may appear deterministic in an unblinded setting due to the periodic balance invoked at the end of each block [17]. Although treatment balance is achieved using the block design, selection bias may occur due to deterministic nature of every even randomization. For example given a three treatment clinical trial with a block size of six where the first five subjects have a treatment assignment sequence of '2,3,1,1,2' then the next assignment of 3 is known and therefore deterministic. Selection bias may be reduced utilizing a variable block design where block size itself is randomly selected, i.e. a permuted block design. Finally, the blocked randomization scheme does not provide restrictions for covariate balance.

Covariate balance and treatment balance may be obtained by using a stratified permuted block design where a permuted block randomization scheme is preformed within each stratum. Strata must be limited for the blocked design since stratifying a permuted block designs permits a maximum of unfilled blocks equal to the additive number of levels within each strata. Multiple unfilled blocks may produce treatment imbalance. In summary, stratified permuted block randomization will create approximate treatment balance in strata; however, imbalance for the total trial may still occur when there are a large number of strata or the block sizes are too large for the number of patients enrolled [18].

A major advantage of the permuted block design is its ease of implementation. Once stratifying factors, block size and number of treatment arms have been determined a schedule of treatment assignment may be produced before the clinical trial begins. The treatment assignment then remains static throughout the course of the trial. A proper analysis that includes all stratifying factors and block can then be performed to ascertain whether treatment effect differences exist.

### Urn randomization

A permuted block design is a non-adaptive procedure or a method of 'fixed' allocation (the probability of treatment assignment is unchanging throughout the course of a trial). Adaptive randomization changes or varies the probability of allocation as the trial progresses. It is a dynamic not a static process for treatment assignments as enrollment accrues. As long as treatments are balanced, participants will have equal probability of assignation. At any accrual point at which an imbalance occurs, the adaptive randomization allocation probability is adjusted so that the probability of assignment is higher for the treatment arm with fewer participants, i.e. treatment imbalances are corrected as the trial's enrollment progresses. We describe an example of an adaptive scheme, the urn randomization process, in the following paragraphs.

The urn design [31] incorporates probabilities of assignment that adapt according to the degree of treatment imbalance. Wei's urn design may be described as follows: an urn contains a set number of balls of two types 1 and 2. Upon randomization of a particular participant a ball is drawn and replaced, if the ball is of type 1 then the participants is assigned treatment 1 and a set number type 2 balls are added to the urn; whereas, if a ball of type 2 is drawn the participant is assigned treatment 2 anda set number of type 1 balls are added to the urn. Thus the composition of the urn is such that the probability of assignment is larger for the treatment type, which has been, selected less often at any point in the trial.

For clinical trials with small sample size, urn randomization forces balance but as the sample size increases the allocation process can be shown to approach that of complete randomization [19]. The urn randomization design is a compromise between complete randomization design and the permuted block design; the probability of correct guess is lower for the urn design compared to the blocked design but is higher than that for complete randomization. The primary focus of urn randomization is that of randomization not balance [9]. However, the urn randomization scheme greatly reduces the probability of treatment imbalance compared to complete randomization whereas treatment imbalance is eliminated in blocked schemes with all blocks filled. There are several difficulties with urn randomization, one being the greater difficulty in logistics of its implementation – it is not a schedule produced in advance; it is a dynamic process. Finally, urn randomization does not always provide restrictions on covariate balance; however, like the simple randomization and permuted block designs, subjects may be stratified on pertinent covariates and assignment may be executed for each level of a stratification factor.

### Covariate adaptive

In the case that several covariates are known a priori to influence outcome, covariate adaptive randomization methodology may be used to force balance over both treatment and prognostic factors [20,21]. The primary role of covariate adaptive randomization unlike simple, blocked and urn randomization is to balance not only treatment totals but also prognostic factor distributions between treatments [9]. Stratified randomization has the same goals of balance as the covariate adaptive scheme; however, while stratified randomization promotes parsimony in selection of stratification factors covariate adaptive methods utilizes all covariates thought to influence response.

Covariate adaptive randomization utilizes the method of minimization by assessing the extent of treatment imbalance incorporating several covariates. Power is maximized in the instance that treatment groups are equal in size. Also, when covariates known to affect outcome are equally distributed across groups the treatment effect estimates will remain unbiased. The covariate adaptive randomization scheme balances total treatment numbers while simultaneously balancing treatment assignments within covariate factors. Minimization allows for a maximum number of covariates to be incorporated into the randomization scheme while stratification requires a minimal number in order to prevent over stratification. Finally, covariate adaptive randomization is a dynamic process; therefore, does not have the ease of implementation inherent in complete, simple or blocked randomization schedules. However, computer programs can be readily written to implement a covariate adaptive scheme.

## Methods

The balancing properties of several randomization techniques (complete, simple blocked, urn and covariate adaptive) were compared using the properties of the CBT and Modafinil for Cocaine Addiction Clinical Trial: a NIDA Phase II study. The trial is a clinical example of the multitude of factors, which must be considered when choosing the most favorable randomization scheme. Properties of the clinical trial include: the principal outcome of interest is continuous (total number of non-use cocaine days), three treatment arms (placebo, 200 mg Modafinil, and 400 mg Modafinil) and several covariates (gender and total days of cocaine use (>10, < = 10) 30 days prior to treatment) known a priori to be highly associated with outcome. Also, the expected gender distribution of the target population is approximately 25% female and 75% male; the expected cocaine days in the target population is 50% for greater than 10 days of cocaine use in the past 30 days and 50% for less than or equal to 10 days of cocaine use in the past 30 days. Other covariates, which may be of interest but are not as strongly associated with outcome, include severity of withdrawal symptoms, severity of depressive symptoms, and presence or absence of Attention Deficit Hyperactivity Disorder (ADHD). The prevalence of comorbidities in the cocaine dependent population is as follows: 15% of cocaine users seeking treatment have adult ADHD, 30% have depression and 50% have high vs. low withdrawal symptoms [22-25]. Finally, based on simple power considerations, the total planned enrollment of subjects is estimated to be 264.

The comparisons of the allocation methods described earlier utilize Monte Carlo simulated data based on the particular properties of this Modafinil protocol. Covariates of gender and past cocaine use were selected on the basis of biological significance and past studies [22,26,27]. Randomization methods were compared with respect to both treatment and covariate imbalance.

Using SAS version 9.0 [28] a random number was simulated and assigned to a treatment group using the randomization procedures described above until a sample of 264 was reached. In order to assess treatment imbalance for each of the randomizations schemes, the range of imbalance was computed for each simulation.

The range of imbalance was computed as the difference between the number of patients in the largest treatment group compared to that in the smallest treatment group. Letting *N*_*k *_equal the number of patients given treatment k where *k *= (1, 2, ..., *K*), the range of imbalance is defined as:

*Range *= max (*N*_1_, *N*_2_, *N*_3_) - min (*N*_1_, *N*_2_, *N*_3_) [20,29]

Comparisons of prognostic factor imbalance across treatments were evaluated for the various random allocation procedures. A population of subjects was generated with the prognostic factor characteristics of gender and low or high previous cocaine use. At each stage of the simulation, a subject was sampled from the population of subjects and assigned a treatment utilizing the various allocation methods. Once a sample size of 264 was reached, the imbalance in the distribution of prognostic factors between treatments for each allocation method was measured and compared for the randomization schemes. For each comparison, one thousand simulations were preformed.

The level of imbalance for treatment groups within the level of a binary prognostic factor was calculated by letting an arbitrary prognostic factor, defined as *X*_*jk*_, have a joint multinomial distribution with index, N, and equal probabilities [20,30].

Then *q*_11 _= *X*_11 _/ (*X*_11 _+ *X*_21 _+ *X*_31_) and *q*_12 _= *X*_12 _/ (*X*_12 _+ *X*_22 _+ *X*_32_) are the proportion of subjects in each factor level on treatment 1. Proportions of subjects in each factor level for each treatment were then calculated. The treatment imbalance for factor 1 is defined as max |*q*_*j*1 _- *q*_*j*2_| where j = 1, ..., 3 treatments. Using this methodology a maximum range of imbalance was recorded for each factor and compared for each allocation procedure.

Finally, simple randomization with stratification was compared with the covariate adaptive scheme with respect to imbalance in non-stratifying factors. Covariate adaptive randomization utilizes the method of minimization by assessing the extent of treatment imbalance incorporating all covariates thought to influence outcome [20]. On the other hand, stratification reduces imbalances in distributions of only a few covariates known to be highly associated with outcome. For the Modafinil trial simulations, simple randomization supplemented with the stratification factors of cocaine use and gender, which are known to be highly associated with outcome, was compared to the adaptive randomization scheme in which minimization factors included all known to be associated with outcome (cocaine use, gender, withdrawal, ADHD presence, and depression). Specifically, subjects were sampled from the cocaine dependent population having the prognostic factor characteristics of gender, low or high previous cocaine use, low or high withdrawal symptoms, presence or absence of ADHD and presence or absence of depression. At each stage of the simulation, a subject was sampled from the population and assigned to a treatment utilizing the various allocation methods.

In order to characterize the covariate balancing properties of these schemes under a smaller sample size assumption, further simulations were conducted given a total sample size of 66. Once a sample size of 66 or 264 was reached, the imbalance in the distribution of prognostic factors of withdrawal, ADHD and depression between treatments for each allocation method was measured and compared for the randomization schemes. That is, the imbalance of three non-stratifying covariates in the simple randomization was compared to the same three minimization factor used for the adaptive scheme. Results were used to assess whether minimization using all five covariates was more advantageous than stratification using two covariates (cocaine use and gender) given these small and moderate sample sizes.

## Results and discussion

### Total treatment balance

**Figure 1:**
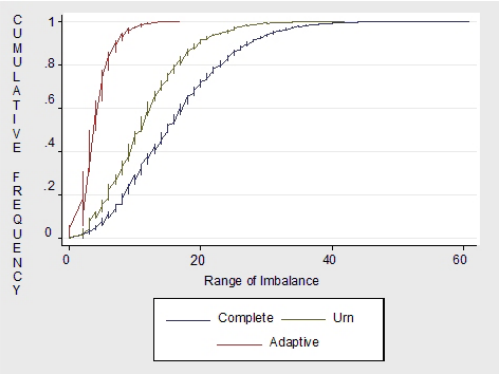
Adaptive (2 covariates), urn and complete randomization (n = 264, treatments = 3, simulations = 1000).

To indicate the degree of treatment imbalance obtained under complete, urn and adaptive randomization, Figures 1 plots the cumulative frequency distribution of the range of imbalance over 1000 simulations. The plot demonstrates the probability of treatment assignment imbalance, which can occur under each randomization scheme. As indicated in the plot, for about 60% of the simulations the range of imbalance was approximately less than 20 for complete randomization, less than 15 for urn randomization and less than 10 for adaptive randomization. Although rare in occurrence, for complete randomization the maximum range of imbalance for all 1000 simulations was 60. Urn randomization had a maximum imbalance of approximately 40 while adaptive randomization has a maximum imbalance less than 20. Simple and blocked randomization were not plotted since they are restricted to produce perfect treament balance.

Table 2 compares complete, urn, adaptive randomization given two minimization factors and adaptive randomization given five minimization factors with respect to the mean and variance of imbalance of total treatment allocation computed over 1000 simulations. Results indicate that the urn randomization scheme has less variability compared to complete randomization with both adaptive randomizations having the smallest variability. Adaptive randomization given 2 minimization factors has the same variability in total treatment balance as adaptive randomization given 5 minimization factors. All schemes show little bias (all three treatment groups have a mean number of subjects of about .333). The simple randomization and blocked randomization are not compared to complete, urn and adaptive randomization because the simple randomization will always produce perfect treatment balance and as long as blocks are filled, blocked randomization will also produce perfect treatment balance.

**Table 2:** Total treatment balance: mean and variance (n = 264, simulations = 1000)

**RANDOMIZATION PROCEDURE**	**TREATMENT**	**E(N_T_(n)/n)**	**VAR(N_T_(n)/n)**
COMPLETE	1	0.3323	0.0013
	2	0.3345	
	3	0.3332	
URN	1	0.3311	0.0007
	2	0.3344	
	3	0.3345	
COVARIATE ADAPTIVE (2 COVARIATES)	1	0.3345	0.0001
	2	0.3352	
	3	0.3305	
COVARIATE ADAPTIVE (5 COVARIATES)	1	0.3351	0.0001
	2	0.3337	
	3	0.3312	

### Covariate balance

In this section, we are interested in comparing whether randomization techniques that balance covariate distributions between treatment arms are useful given a moderate size clinical trial. As mentioned previously, using stratification with simple or blocked randomization will produce perfect covariate balance between treatment arms. However, selection of stratifying factors must be minimized. On the other hand, covariate adaptive randomization uses all covariates known a priori to influence treatment to produce balance. However, covariate adaptive randomization will produce slightly less balance in covariate distributions between treatment arms.

**Figure 2:**
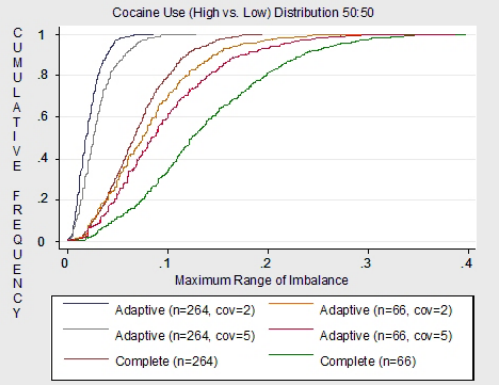
Complete and adaptive randomization: cocaine covariate imbalance (treatments = 3 simulations = 1000).

**Figure 3:**
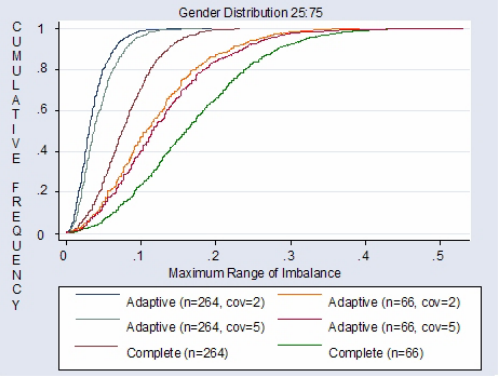
Complete and adaptive randomization: gender covariate imbalance (treatments = 3 simulations = 1000).

Using no stratification and simple, blocked or urn randomization will produce identical amounts of covariate imbalance as complete randomization since these randomization techniques focus on balances in the total number of subjects assigned to treatment arms and not balances of prognostic factors between treatment arms. Whether complete, simple, permuted block or urn randomization is used to illustrate covariate balance is negligible. For convenience, comparisons of covariate balance are illustrated in figures 2 and 3 using complete randomization (with no stratification) versus covariate adaptive randomization. The results are used to demonstrate that given a sample size of 264, randomization techniques that focus on covariate balance may not be necessary. If a technique is utilized to minimize covariate imbalance, it is best to use a simplistic technique such as stratification rather than more complex techniques such as covariate adaptive randomization e.g. minimization. The results of this study, in general, apply to clinical trials of moderate to large sample sizes. Therefore, a small sample size of 66 may require more stringent restrictions on covariate balance.

Figures 2 and 3 plot the cumulative frequency distribution of cocaine use (distributed 50:50) imbalance and gender (distributed 25:75) imbalance between treatment arms respectively. Both the cocaine covariate and the gender covariate demonstrate least imbalance given that an adaptive randomization scheme was utilized compared to a non-stratified complete randomization scheme. Also, fewer imbalances are demonstrated for the adaptive scheme with only two minimizing factors compared to the scheme with five minimizing factors. This is an interesting finding, further emphasizing the importance of selecting only the most powerful factors when considering restriction on randomization.

As expected, covariate imbalance for a sample size of 264 is much less than covariate imbalance for a sample size of 66. Specifically, complete randomization given a sample size of 264 and adaptive randomization given a sample size of 264 have small imbalance overall. Although complete randomization does demonstrate a higher probability of imbalance than adaptive randomization both schemes have very small maximum imbalances. Therefore, complete randomization may be expected to be as effective in balancing covariate distributions between treatment arms as adaptive randomization given a moderate to large sized clinical trial.

Overall results demonstrate little variability in covariate imbalance between randomization procedures given a moderate to large clinical trial. Therefore in the case of the Modafinil trial, which has a sample size of 264, randomization restrictions that control for covariate imbalance may not be necessary, and certainly if restrictions are to be imposed, then stratification on cocaine use and gender alone is the most reasonable strategy. However, results will vary for smaller trials [13] thus illustrating the importance of assessing the properties of each clinical trial independently in order to choose the most optimal scheme.

In order to demonstrate that a small sample size of 66 may require more stringent restrictions on covariate balance compared to a sample size of 264, the ratio of imbalance given the two sample sizes of 66 and 264 is illustrated in Table 3. In general, results of the covariate imbalance indicate a 1.5 to 3 times increase in covariate imbalance given a sample size of 66 compared to a sample size of 264. However, for covariate adaptive randomization, for both the 2 factor and 5 factor versions, there is little difference in treatment imbalance within gender for a sample size of 66 or 264. This may be due to the unequal ratio of males to females (25:75). Therefore, covariate adaptive randomization may be most appropriate in situation of equal distributions of covariates. Since this is an unexpected finding in the study, future simulations may need to be conducted varying covariate distribution and sample size to clarify the relationship between varying covariate distributions and balancing properties on covariate adaptive randomization.

**Table 3:** Chart of the ratio of imbalance given the two sample sizes of 66 and 264

	**Ratio of Imbalance (n = 66)/(n = 264)**	
**RANDOMIZATON PROCEDURE**	**cocaine (50:50) ratio**	**gender (25:75) ratio**

COMPLETE OR SIMPLE	1.99	2.50
STRATIFIED SIMPLE		
URN	3.04	1.53
STRATIFIED URN	1.90	2.80
BLOCKED	1.56	2.65
STRATIFIED BLOCKED		
COVARIATE ADAPTIVE (2)	2.38	0.80
COVARIATE ADAPTIVE (5)	2.21	1.20

Simple randomization with stratification factors of cocaine use and gender was compared to adaptive randomization with five minimization factors (cocaine use, gender, withdrawal, ADHD and depression). The three covariates, which were not used as stratifying factors, were then compared for differences in imbalance under the simple randomization and the adaptive randomization scheme. The purpose of this comparison was to determine the necessity of controlling for all covariates thought to be associated with outcome in the randomization process e.g. whether covariate adaptive randomization using all covariates is necessary or whether a simple scheme using a few covariates as stratifying factors will suffice for moderate sample sizes.

**Figure 4:**
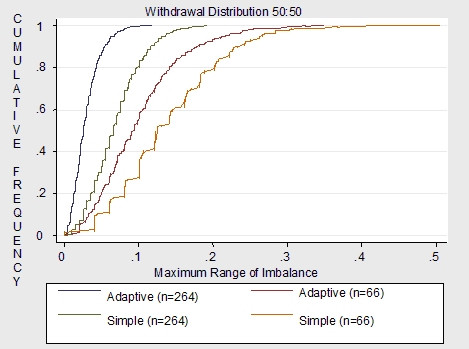
Non-stratifying factors: stratified simple vs. adaptive (5 covariates) for n = 66 and n = 264.

**Figure 5:**
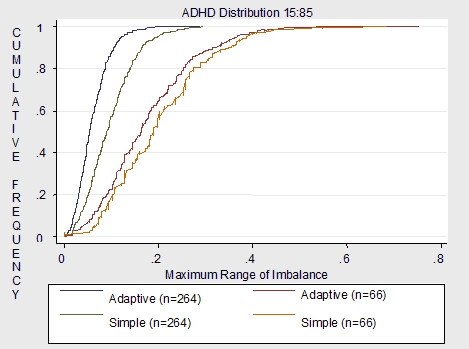
Non-stratifying factors: stratified simple vs. adaptive (5 covariates) for n = 66 and n = 264.

**Figure 6:**
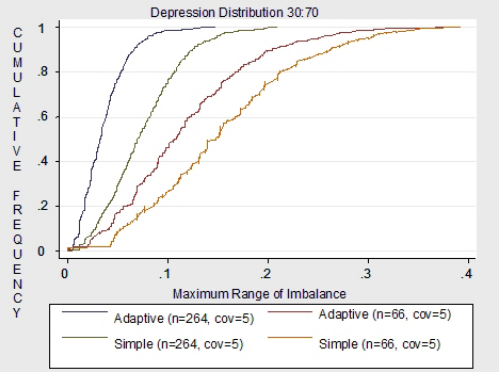
Non-stratifying factors: stratified simple vs. adaptive (5 covariates) for n = 66 and n = 264.

Figures 4, 5 to 6 demonstrate the imbalance produced by the non-stratifying factors of simple randomization compared to the imbalance of the same factors used for minimization in the adaptive randomization scheme. Overall results demonstrate much more imbalance given a sample size of 66 compared to a sample size of 264. Given a sample size of 66, simple randomization which does not stratify by ADHD, withdrawal and depression is much more imbalanced than the adaptive scheme which uses all five covariates as minimization factors. Also, imbalance varies dependent on the distribution of the covariate. For withdrawal, which is distributed 50:50, the adaptive scheme has a smaller maximum of imbalance than the simple randomization. For the depression covariate, which is distributed 30:70, and the ADHD covariate, which is distributed 15:85, the maximum covariate imbalance is the same for simple randomization and the covariate adaptive scheme.

Given a sample size of 264, results demonstrate that imbalance is similar for both the non-stratifying factors of simple randomization and the minimization factors of the adaptive scheme. Although the adaptive scheme had less probability of imbalance overall compared to the simple randomization, for all covariates the maximum imbalance for both schemes was around .2. Therefore given a moderate to large sample sizes, the simple scheme may be as justified for use as the adaptive scheme.

In summary, using the properties of the Modafinil clinical trial for purposes of simulation demonstrated large treatment imbalance in complete randomization with less imbalance for the urn or adaptive scheme. Randomization methods, such as the urn or adaptive, have smaller treatment imbalance as demonstrated by the low variability of treatment allocation imbalance. For all randomization schemes, covariate imbalance between treatment arms is small which may be due to the large size of the Modafinil trial (n = 264). Covariate imbalance may be better-controlled using stratification or adaptive baseline randomization for trial of small size but is less of an issue for this Modafinil trial. An optimal randomization scheme for the Modafinil trial should focus on treatment balance with less focus on covariate balance. Specifically, a simple or blocked scheme with a one or two stratification factors will be as effective as the more complex urn or highly adaptive schemes.

### Randomization techniques and statistical analysis

Stratification was defined above as a technique, which partitions participants into mutually exclusive subsets defined by initial covariates thought to influence response in order to reduce accidental bias. An advantage of stratification in the design stage is an increase in power: the ability to detect a difference in treatment groups when a difference truly exists. However, unstratified randomization in moderate to large trials is approximately equivalent with respect to power to that of stratified randomization [3]. Therefore, for moderate to large clinical trials stratified randomization versus a stratified analysis following a non-stratified randomization may be negligible. However, given a stratified randomization, a stratified analysis should follow. Studies of 100 participants demonstrate that stratified randomization with adjusted model-based population analyses increases power by up to 12% compared to no stratification with non-adjusted analysis [11,13,15].

A stratified block design utilizes a block randomization scheme within each stratum, which not only minimizes imbalance among treatment but also minimizes imbalance over covariates that may be related to the response variable. However, a stratified permuted block design has a higher chance of unfilled blocks than a permuted blocked scheme alone; hence it is important to limit the number of strata to the most important covariates to minimize the risk of treatment imbalance. Hallstrom and Davis [18] demonstrate that stratified permuted block randomization will create approximate treatment balance in strata; however, imbalance for the total trial may still occur when there is a large number of a stratum or the block sizes are too large for the number of participants enrolled.

The principal of blocking is to increase the power for treatment comparisons by dividing experimental units into homogenous strata and then pooling the treatment group differences over blocks [17]. In the instance of a clinical trila where patients are gradually accrued over time, participants may be time heterogeneous. Therefore, incorporating blocking within the analysis should provide a more powerful test of the treatment effect. Data may be examined for the existence of an intrablock correlation (participant responses within blocks may be positively correlated because they are recruited closer in time) and a block-stratified analysis may be required dependent on the existence of an intrablock correlation due to time heterogeneity [11,17]. In the instance of a positive intra-block correlation, an analysis ignoring block will be conservative (have higher Type II error).

In the statistical literature, discussions of analysis given that urn randomization was utilized in the design stage of the study promote the use of permutation tests [7,19]. The effect of ignoring the randomization in the presence of heterogeneity is not as easily illustrated as that of blocked randomization. Additionally, tests based on a permutation model for the urn design may differ substantially from tests based on a parametric or population model (normal distribution, variance homogeneity, etc.). Due to the inherent time heterogeneity expected in a clinical trial as well as the difficulty in quantifying the effect of urn randomization on outcome, the proper permutation tests whose variance account for randomization restrictions are suggested over the use of statistical tests based on population models [31].

An example of the effect of ignoring the randomization in the analyses is the extra corporeal membrane oxygenation (ECMO) versus standard therapy clinical trial for infants with persistent pulmonary hypertension [32]. The ECMO study was designed using *outcome *adaptive randomization (not covered in this paper), i.e. the randomized play the winner (RPW) rule of Zelen [33]. Wei [19] demonstrated the differences in significance that can occur when the randomization is ignored in the analysis. Conclusions of the analyses of the ECMO study were that the statistical significance of treatment effect was inflated when the randomization design was ignored in the analysis.

Covariate adaptive randomization utilizes the method of minimization assuring that treatment arms are balanced within various strata of predefined covariates [20,21]. A disadvantage of covariate adaptive randomization is the complexity introduced into the analysis. Taves promoted the use of ANCOVA for analysis where all covariates used as minimization factors are also used in the analysis [21]. However, the correct statistical methods for covariate adaptive randomization of analysis are still a conundrum in statistical sciences [3,34,35]. Along with ANCOVA, permutation tests which take into account the particulars of the adaptive randomization scheme have been suggested for analysis.

In conclusion, given that all assumptions are met the type of randomization scheme may be ignored in the analysis and the population model may be used as a method of inference. However, assumptions based on population models are tenuous at best. In the case of accrual in a clinical trial where time heterogeneity of outcome is likely, population-based tests may not be valid. Permutation tests on the other hand assume nothing about the data except that participants were randomized. Under the permutation model of inference, restrictions of the randomization scheme may be incorporated into the analysis. Much of the statistical literature on randomization and analysis suggests incorporating a permutation method of analysis for a blocked design in the presence of intrablock correlation, for the urn design where time heterogeneity is not as easily assessed and for covariate adaptive design where minimization complicates the analysis [3,6,7,9-11,18,31,36]. In summary, failure to account for restriction in analysis may result in conservative tests of significance. This aspect of clinical trial design and analysis requires further investigation, particularly implications for substance abuse trials.

## Conclusion

The purpose of the article was to review several randomization techniques used in practice in order to aid the substance abuse researcher in designing and analyzing a RCT. Complete, simple, blocked, urn and covariate adaptive designs were compared with respect to balancing properties and their implications on statistical analysis. Complete randomization minimizes selection bias; however, has the maximum amount of treatment imbalance. As long as blocks are filled, blocked randomization eliminates treatment imbalance but may be more deterministic due to periodic balancing properties. Urn randomization is less deterministic than blocked randomization and has lower probability of imbalance in small sample sizes than complete randomization. Covariate adaptive randomization not only minimizes overall treatment imbalances but also minimizes the imbalances in prognostic factor distributions.

Balancing properties of the randomization schemes were further discussed using a clinical application, The Cognitive Behavioural Therapy (CBT) and Modafinil for Cocaine Addiction Clinical Trial. Simulation studies using the properties of the Modafinil RCT demonstrated large treatment imbalance in complete randomization; while, urn randomization scheme demonstrated small treatment imbalance and covariate adaptive randomization demonstrated the least imbalance.

For all randomization schemes, covariate imbalance between treatment arms was small with little variation between adaptive schemes, stratified schemes and unstratified schemes given that sample size was moderate to large. Given the specific properties of the Modafinil trial, the particular randomization scheme chosen may want to focus on treatment balance restrictions rather than a covariate balance restraints. However, the substance abuse researcher should note that results might vary for trials of smaller sample size, more stratification factors, or a different number of treatment arms [13].

Finally, tests of statistical significance for randomized studies can be based on either a permutation model or a population model. Whereas a permutation model requires no assumptions regarding the origin of the study participants or the distribution of their responses a population model (most often used in clinical studies) assumes that participants were sampled at random from a homogenous population and their responses follow a common distribution [7]. Under the assumption of a homogenous or unchanging population, the method of randomization may be ignored in the analysis; however, one should not automatically assume homogeneity for most clinical trials. Permutation methods of analysis have been suggested for the urn and covariate adaptive schemes. The analysis becomes more complicated by these restrictions placed on the probability of assignation [3,9,10,31]. Furthermore, whether or not a permutation or population model is assumed, covariates used for randomization techniques such as stratification or minimization should be accounted for in the analysis stage of the study [9].

There are many issues associated with the design of clinical trials in substance abuse research. Randomization represents one of the many design features typically under consideration. *While the use of adaptive randomization designs and urn allocation methods has become increasingly popular, it is noteworthy that simply conceived stratification designs with simple or permuted blocked randomization schedules within strata continue to offer an efficient, as well as easily implemented alternative*. There are many factors to consider in designing such trials, and there may be too quick a tendency to try to correct for every conceivable confounding baseline factor. Parsimony in baseline factor selection coupled with simple stratification and randomization offer the substance abuse clinical trialist some straightforward and powerful design tools.

Finally, *randomized *clinical trials are the gold standard of evidence-based medicine. The top tier of the hierarchy of evidence-based medicine includes clinical trials that are less vulnerable to bias, are generalizable and are valid [1].  Randomization techniques, when used appropriately, serve to lessen the vulnerability of a study to bias and provide validity for tests of statistical significance [3].  Therefore, increasing the knowledge base of randomization techniques for substance abuse clinical trials promotes strong study design, which in turn, promotes confidence that the outcomes observed are due to the treatment under study.

## Competing interests

The author(s) declare that they have no competing interests.

## Appendices

### Appendix 1: Complete randomization

Complete randomization is often described as 'a toss of a fair coin' whereby a participant is assigned to treatment 1 if the toss of a coin produces a head and is otherwise assigned to treatment 2. Probabilistically, complete randomization is defined letting T_1_, ..., T_n _be a sequence of random treatment assignment where i = 1 to n. Then T =1 when a participant is assigned to treatment 1 and T = 0 when a participant is assigned to treatment 2. Complete randomization assumes that T_1_, ..., T_n _are independent identically distributed Bernoulli random variables where P(T_i _= 1) = 1/2.

Complete randomization may also be generalized to clinical trials with three treatment arms. Letting T_1_, ..., T_n _be a sequence of random treatment assignments assume that each of the n independent, identical trials have an outcome in any three treatments. Then T_i _= j; j = 1, ..., 3 if participant i has outcome in treatment j. T_1_, ..., T_n _are independent identically distributed (i.i.d) random variables with P(T_i _= j) = 1/3; j = 1, 2, 3; i = 1, ..., n. For K>2 groups, complete randomization becomes 'a simple multinomial probability generator' [3,16].

### Appendix 2: Permuted block as an example

A block is defined as a pre-specified number of subjects who are randomized to an equal proportion of treatment assignments [16,17]. Specifically, the block design consists of M blocks containing n = N/M participants where N is the total sample size. Within each block given two treatment arms, n/2 participants are assigned to treatment 1 and n/2 are assigned to treatment 2. A random allocation rule is utilized in each block to ensure balance throughout the course of the trial. In other words, at any M stage in the course of a trial allocation (at the end of each block of assignments) perfect balance occurs. Also, the maximum amount of imbalance that can occur at any point in time during a trial is limited to n/2. Finally, block designs are easily generalized to k>2 treatments. For three treatments, the block design may be described as follows: the block design consists of M blocks containing n = N/M participants; within each block, n/3 participants are assigned to each of the j = 1, ..., 3 treatments.

### Appendix 3: Urn randomization

The urn design [31], a form of adaptive randomization, incorporates probabilities of assignment that adapt according to the degree of treatment imbalance. Wei's urn design may be described as follows: an urn contains α balls of two types 1 and 2. Upon randomization of a particular participant a ball is drawn and replaced, if the ball is of type 1 then the participants is assigned treatment 1 and β type 2 balls are added to the urn; whereas, if a ball of type 2 is drawn the participant is assigned treatment 2 and β type 1 balls are added to the urn. Thus the composition of the urn is such that the probability of assignment is larger for the treatment type, which has been, selected less often at any point in the trial. For two treatment arms we can let N_1 _and N_2 _be the proportion of participants randomized to treatment arm 1 and 2 out of the total participants randomized at any point in the trial. F_n _is defined as the set of treatment assignments which have been allocated at any n stage of the randomization process, F_n _= {T_1_, ..., T_n_}. F_j-1 _may then be defined as the set of treatment assignments which have been allocated previous to the current treatment to be assigned where *T*_*j *_is the current treatment to be assigned.

The urn design is denoted UD(α, β) and has the following allocation rule:

; where for the first treatment assignment P(T_1_/F_0_) = 1/2. For UD(0, 1) an urn that contains zero balls at the beginning of the study and adds one ball after each assignment, the allocation rule simplifies to . For example, the probability of the 51^st ^assignment to treatment two given that 22 out of the first 50 assignments were assigned treatment two is . Therefore for the UD(0, 1) design there is a 56% chance that the next assignment will be treatment 2 and a 44% chance that the next assignment will be treatment 1 given that 22 out of the first 50 assignments where to treatment 2.

Wei's UD(0,1) design may be generalized to three treatment groups as follows: for the UD(α, β) design the urn contains α balls that represent each treatment initially. Then β balls are added to the urn for each other treatment after each assignment [16]. The probability that the jth assignment is to treatment i given the previous j-1 assignments when UD(0, 1) is as follows:  where i = 1,2,3 represent each of the three treatment arms.

### Appendix 4: Covariate adaptive randomization

Covariate adaptive randomization can be described for three treatment arms letting x_ij _equal the number of subjects already assigned to treatment j (j = 1, ..., k) who have the same level of prognostic factor i (i = 1, ..., p) as the subject to be assigned currently [20,37]. The change in balance allocation when a subject is assigned treatment k can then be represented as follows:



A function of the x^k^_ij_, such as the range or variance, may then be defined that measures the imbalance overall prognostic factors given that the new subject was assigned treatment k. Finally, the treatment assignment which minimizes the imbalance can be assigned with probability greater than 1/3.

## Authors' contributions

SH carried out the Monte Carlo simulation studies and drafted the manuscript.

RW participated in the statistical theory of the study, added to the manuscript and edited the manuscript.

RM participated in the clinical applicability of the study and edited the manuscript.

**Table 1:** Illustration of patient numbers by treatment with 3 levels and covariate with 2 levels

	Factor 1		
Treatment	1	2	

1	X_11_	X_12_	
2	X_21_	X_22_	
3	X_31_	X_32_	
			N
